# Comparison of the Antibacterial Effect of Sodium Hypochlorite and Aloe Vera Solutions as Root Canal Irrigants in Human Extracted Teeth Contaminated with Enterococcus Faecalis

**Published:** 2014-03

**Authors:** S. Sahebi, N. Khosravifar, M. SedighShamsi, M. Motamedifar

**Affiliations:** a Dept. of Endodontics, School of Dentistry, Shiraz University of Medical Sciences, Shiraz,Iran; b Postgraduate Student of Radiology, Dept. of Radiology, School of Dentistry, Shiraz University of Medical Sciences, Shiraz,Iran; c Dept. of Microbiology, School of Medicine, Shiraz University of Medical Sciences, Shiraz,Iran

**Keywords:** Aloe Vera, Sodium hypochlorite, Enterococcus faecalis, Root canal irrigants

## Abstract

**Statement of Problem:** The main purpose of a root canal treatment is to eliminate the bacteria and their products from the pulp space. Sodium hypochlorite has excellent antibacterial properties, but also some negative features.

**Purpose:** The aim of the present study is to compare the antimicrobial effect of Aloe Vera solution with sodium hypochlorite on E.faecalis in the root canals of human extracted teeth.

**Materials and Method:** Sixty human extracted single rooted teeth were selected for this in vitro study. The teeth recruited in this study had no cracks, internal resorption, external resorption and calcification. Enterococcus faecalis was injected in the root canals of all teeth. The teeth were then divided into three groups randomly. Each group consisted of 20 teeth that were all rinsed with one of the following solutions: sodium hypochlorite 2.5%, Aloe vera and normal saline. Subsequent to rinsing, root canals of all teeth were sampled. The samples were cultured and growth of the bacteria was assessed after 48 hours. The number of colonies of the bacteria was then counted.

**Results:** The difference between the inhibitory effect of Aloe vera and normal saline on E.faecalis was not significant according to independent t-test (p= 0.966). The inhibitory effect of sodium hypochlorite on E.faecalis was much greater than that of Aloe vera and normal saline (p< 0.001).

**Conclusion: **Aloe vera solution is not recommended as a root canal irrigator, but future studies are suggested to investigate the antibacterial effect of Aloe vera with longer duration of exposure and as an intra canal medicament.

## Introduction

Bacteria and their products play the main role in the initiation and exacerbation of pulp and periapical diseases. In 1894, Miller introduced the role of bacteria in the pathogenesis of pulp diseases for the first time [1]. Therefore, the main purpose of a root canal treatment is to eliminate the bacteria and their products from the pulp space. It has been figured out that the most probable cause of the failure in root canal treatments is the presence of oral bacterial flora in the apical portion of the root canal [2] . In a research done by Sundqvist on necrotic human teeth, it was concluded that apical periodontitis only occurs in teeth containing bacteria in their root canals [3] . Enterococcus faecalis (E. faecalis) is a part of the normal flora of the oral cavity that can be detected in small numbers in the treated root canals, but in large numbers in teeth with endodontic treatment failure [4] .

In 2001, Hancock et al. showed that the most prevalent bacterial strain detected in teeth with endodontic treatment failure is Enterococcus faecalis [5]. In root canal therapy, in order to eliminate the bacteria derived irritating factors, mechanical debridement of the pulp space, alone, is not sufficient and should always be accompanied by chemical debridement. Various irrigating solutions have been used to fulfill this aim, of which sodium hypochlorite is the most common due to its several desirable properties [6]. However, sodium hypochlorite has some negative features such as unpleasant taste and odor, allergic reactions in some patients, risk of sodium hypochlorite accident, toxicity and metal corrosion [7]. In the recent years, the use of herbal products as root canal disinfectants has been widely investigated in endodontics because of their efficiency, safety and accessibility [8] . Unlike sodium hypochlorite, Aloe Vera extract not only owns anti- inflammatory properties, but also stimulates dental pulp cell proliferation, differentiation and extracellular matrix mineralization [9]. Aloe Vera (Aloe barbadensis Miller) is a kind of plant that is well known for its numerous biologic and therapeutic functions such as wound healing, hypoglycemic effects, anti inflammatory and immune-modulation features and also antimicrobial properties [10]. It has been proven in several studies that Aloe Vera shows considerable antimicrobial activity against various species such as Streptococcus pyogen, Enterococcus faecalis, Candida albicans and staphylococcus aureus [9-10]. The purpose of the present study is to evaluate the antimicrobial effect of Aloe Vera solution on E.faecalis in the root canals of human extracted teeth and also to compare this effect with sodium hypochlorite solution.

## Materials and Method

***Initial preparation of the teeth***

60 human extracted teeth were collected from the dental clinics in Shiraz. All teeth were single rooted and only the ones that did not have conditions such as cracks, internal and external resorption and calcification were included in this experimental study. In order to assimilate the conditions of the teeth, the crowns were dissected at the level of cemento-enamel junction(CEJ) and the root length of the teeth were shortened to 13-14 mm. An endodontic K- file number 15 (Mani; Japan) was inserted in the root canal of each tooth and moved in the apical direction until the tip of the file was observed through the root apex. 1 mm less than the distance passed by the K- file was considered as the working length of each tooth. Apical portion of all teeth was enlarged with K-files (number 15 and 20) according to their working lengths. While preparing the teeth, their root canals were rinsed with normal saline. In order to prevent leakage of bacteria and irrigating solutions from the root apices, all teeth were mounted vertically in self-cure acrylic resin. Subsequently, all teeth were sterilized via an autoclave machine in 121°C and 15 PSI pressure for 15 minutes. A sterility checking test was performed for all teeth.

***Microbiology procedures***

**Figure 1:**
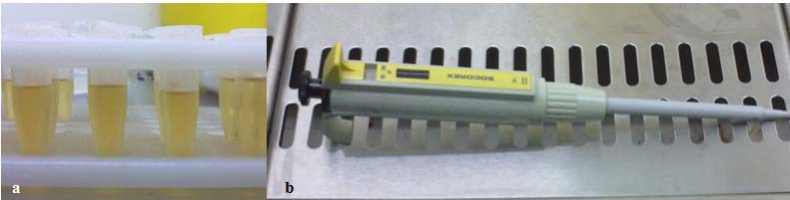
**a** Eppendorf tubes containing TSB solution  **b** Sampler tool used for both contaminating and sampling of the teeth

30 grams Tryptic Soy Broth (TSB) powder (Difco Laboratories, Detroit, Mich.) was dissolved in 1 liter distilled water to prepare 30 g/l TSB solution. 990 microliters of the solution were transferred to each Eppendorf tube (Figure 1a). Also, 40 grams MHA (Mueller Hinton Agar) powder (Oxoid Ltd., London, England) were dissolved in 1 liter of  distilled water to prepare 40 g/l MHA solution. MHA solution was transferred to culture plates. The plates were placed inside a refrigerator. Enterococcus faecalis standard strain ATCC 11700 was prepared in the bacteriology laboratory of Shiraz, School of Medicine. Bacterial count was 1.5×10^ 8 ^colony forming units/ milliliters (CFU/ml). Considering the root canal diameters, 10-20 microliters of the bacterial suspension was injected in the root canal of each tooth by means of a sampler tool (Figure 1b). Contaminated teeth were kept moist in order to prevent evaporation of bacterial suspension from the root canals. The teeth were incubated in 37°c for 48 hours.

After 48 hours, to ensure the bacteria had grown in all teeth, each root canal was sampled. The samples were transferred to MHA plates. Positive culture after 48 hours revealed that all of the root canals were contaminated with E.faecalis.

**Figure 2:**
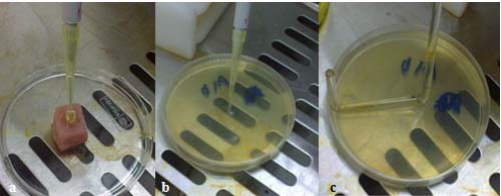
**a** Sampling of the irrigating solution  **b** Transferring samples to MHA culture plates  **c** Spreading of the bacteria with the L- shaped tube

***Irrigation of root canals with different solutions***

Aloe Vera gel was extracted from the plant’s leaves at the animal research center of Shiraz School of Medicine. The leaves of the plants were washed with distilled water and the surfaces of the leaves were disinfected with 70% ethyl alcohol. After cutting and opening the leaves, the fresh pulp was collected and homogenized. 80 grams of the gel were dissolved in 20 milliliters distilled water to prepare 80% Aloe Vera solution.In order to prepare 2.5% sodium hypochlorite solution for the present study, 5% home bleach (Active, Iran) was diluted with distilled water in equal proportions.Normal saline used in this study (Samen, Mashhad, Iran) was sodium chloride 0.9%. Using block randomization, contaminated teeth were randomly divided into three groups: the first group consisted of 20 teeth having root canals prepared according to their working lengths with K-files 25, 30 and 35. While preparing each tooth, 8 milliliters of 2.5% sodium hypochlorite solution was used as the canal irrigator in the following way: before the insertion of each K file into the canal and after instrumentation with the last file (number 35), the root canal was rinsed with 2 milliliters of the solution. For the rinsing procedure, disposable syringes with 22 gauge needles (Avapezeshk; Iran) were used and the duration of rinsing was 15-20 minutes for each tooth. Then each tooth was rinsed with 4% sodium thiosulfate in order to neutralize sodium hypochlorite solution. The second group consisted of another 20 teeth that were rinsed with Aloe Vera solution in exactly the same way described for the first group and then irrigation was washed out with normal saline. Finally, the third group had 20 teeth that were rinsed with normal saline in the same way for the other two groups.

***Final Sampling***

10 microliters of the irrigating solution that existed at the canal entrance of each tooth was sampled with a disposable syringe (Avapezeshk, Iran) and transferred to an Eppendorf  tube that already contained 990 microliters TSB solution. Therefore, the concentration of the solution contained within the Eppendorf tube reached 1/100. 10 microliters of 1/100 solution was transferred to another tube containing 990 microliters TSB solution to reach the concentration of 1/10000 of samples (Figure 2a). 10 microliters of 1/10000 solution was transferred to each MHA plate (Figure 2b). The bacteria were spread over the culture plates by means of an L-shaped tube (Figure 2c).

Each culture plate was incubated at 37°c for 48 hours. After 48 hours, bacterial colonies were counted in MHA plates, each one representing one of the teeth. Data obtained by counting of the bacterial colonies were analyzed by means of one-way-ANOVA and t-test using the statistical software SPSS PASW (predictive analytics software, version 18).

## Results

Data were entered in the statistical software SPSS PASW 18. Mean value and standard deviation were calculated for each group. The results were as follows: the three groups were compared to each other via independent t-test. Aloe Vera and normal saline showed no significant difference in antibacterial effect on E.faecalis (p= 0.966) while sodium hypochlorite had a significant ly more antimicrobial effect than the other two groups (p< 0.001) (table 1).

**Table 1:** Mean value and standard deviation of the bacterial colony counts in each group.

	**The Aloe Vera Group**	**The Normal saline Group**	**The Sodium hypochlorite Group**
Mean value	2.21 × 10^ 8 ^	2.27 × 10^ 8 ^	0
Standard deviation	0.98 × 10^ 8 ^	0.95 × 10^ 8 ^	0

## Discussion

An ideal irrigating solution should possess maximal antimicrobial and tissue solving properties and minimal toxic effects [11]. Sodium hypochlorite has long been used as a root canal irrigator because of its enormous antibacterial activity [12]. Several studies have revealed that the antibacterial effect of sodium hypochlorite is much greater than normal saline and other root canal irrigating solutions [7]. Aloe Vera is a kind of plant that its leaf is composed of two major parts. The outer green rind contains vascular bundles and the inner colorless part, named as aloe gel, includes the parenchymal cells which contain viscous clear liquid [10]. The aloe gel consists of 99.5% water. The remaining solid material includes mineral, vitamins, enzymes, polysaccharides, phenolic compounds and organic acids [10]. It has been proved to have multiple therapeutic and antimicrobial properties, especially on Enterococcus faecalis [8, 10, 13-15]. Furthermore, it has been successfully used for decontamination of gutta percha [16]. In the present in vitro study, Enterococcus faecalis which is a gram positive bacterial strain was used to evaluate the antibacterial effect of irrigating solutions due to its proved resistance to antimicrobial agents [4]. Several studies have shown that Aloe vera extract, especially acts against gram positive bacteria [17]. The antibacterial activity of Aloe Vera is related to anthraquinones which are phenolic derivatives [10]. According to Sureshchnadra et al., Aloe Vera extract is effective against Enterococcus faecalis in agar medium [14]. However, agar diffusion tests have some limitations, including the influence of substrate pH, incubation period and diffusion capacity of the drug on the test results [14]. Bhardwaj et al. assessed antibacterial activity of Aloe Vera gel as long as 1, 3 and 5 days. Aloe Vera showed good antibacterial activity just at the first day of incubation. They noted that Aloe Vera had 75 potentially active constituents such as vitamins, enzymes, minerals, sugars, lignin, saponins, salicylic acids and amino acids which were possible reasons for its antimicrobial action [18]. We evaluated the antibacterial activity of Aloe Vera for a short-time usage (15 to 20 minutes).

Despite previous studies, the results of the current study show that Aloe vera and normal saline have a similar inhibitory effect on E.faecalis which is far less than the antimicrobial effect of sodium hypochlorite. Several factors could be considered for this outcome: first, a period of 15- 0 minutes might not be sufficient for Aloe vera to apply its inhibitory effect against E.faecalis. However, no studies were available to suggest a specific duration for Aloe vera to act as an antimicrobial agent. Second, tooth structures themselves might lessen the antibacterial effect of Aloe vera solution. Lawrence et al. stated that microbial toxicity of Aloe Vera is related to the site and number of hydroxyl groups in the phenol groups [17]. Hydroxyl groups are responsible for alkalinity and antibacterial action of calcium hydroxide but its effect is relatively neutralized by dentin buffering action. Therefore, the antibacterial activity of Aloe Vera was suppressed by this mechanism. Third, the gel-like consistency of Aloe Vera could cause limited flow of the substance through the irregularities of the root canal system.

Aloe Vera polysaccharides are also competent enhancing agent for innate immunity and eliminate the oxidative injury in oral ulcers of animals [19] . Further studies are recommended to evaluate its anti inflammatory effect on periapical tissues and reduction of pain.

## Conclusion

According to the results of the present study, Aloe Vera solution is not recommended as a root canal irrigator, therefore, future studies are recommended to investigate the antibacterial effect of Aloe Vera with longer  duration  of  exposure and also as an intra canal medicament.
